# VDR Agonist Prevents Diabetic Endothelial Dysfunction through Inhibition of Prolyl Isomerase-1-Mediated Mitochondrial Oxidative Stress and Inflammation

**DOI:** 10.1155/2018/1714896

**Published:** 2018-04-15

**Authors:** Meijin Zhang, Liming Lin, Changsheng Xu, Dajun Chai, Feng Peng, Jinxiu Lin

**Affiliations:** ^1^The First Clinical Medical College, Fujian Medical University, Fuzhou, Fujian, China; ^2^Department of Cardiology, Affiliated Hospital of Putian University, Putian, Fujian, China; ^3^Fujian Provincial Institute of Hypertension, the First Affiliated Hospital, Fujian Medical University, Fuzhou, Fujian, China; ^4^Department of Cardiology, the First Affiliated Hospital, Fujian Medical University, Fuzhou, Fujian, China

## Abstract

**Background and aim:**

Upregulation of prolyl isomerase-1 (Pin1) protein expression and activity was associated with the pathogenesis of diabetic vasculopathy through induction of endothelial oxidative stress and inflammation. Moreover, VDR agonist protects against high glucose-induced endothelial apoptosis through the inhibition of oxidative stress. We aimed to explore the effects of the VDR agonist on diabetes-associated endothelial dysfunction and the role of Pin1 in this process.

**Methods:**

Streptozocin-induced diabetic mice were randomly treated with vehicle, VDR agonist (10 *μ*g/kg/d, i.g., twice a week), or Pin1 inhibitor, Juglone (1 mg/kg/d, i.p., every other day), for eight weeks. In parallel, human umbilical vein endothelial cells (HUVECs) exposed to high-glucose condition were treated with 1,25-dihydroxyvitamin D_3_ and Juglone or vehicle for 72 hours. Organ chamber experiments were performed to assess endothelium-dependent relaxation to acetylcholine. Circulatory levels of Pin1, SOD, MDA, IL-1*β*, IL-6, and NO in diabetic mice, Pin1 protein expression and activity, subcellular distribution of p66Shc, and NF-*κ*B p65 in high glucose-cultured HUVECs were determined.

**Results:**

Both VDR agonist and Juglone significantly improved diabetes-associated endothelial dysfunction and reduced high glucose-induced endothelial apoptosis. Mechanistically, the circulatory levels of SOD and NO were increased compared with those of vehicle-treated diabetic mice. Additionally, Pin1 protein expression and activity, p66Shc mitochondrial translocation, and NF-*κ*B p65 in high glucose-cultured HUVECs were also inhibited by VDR agonist and Juglone. Knockdown of VDR abolished the inhibitory effects of VDR agonist on high glucose-induced upregulation of Pin1 protein expression and activity.

**Conclusions:**

VDR agonist prevents diabetic endothelial dysfunction through inhibition of Pin1-mediated mitochondrial oxidative stress and inflammation.

## 1. Introduction

Atherosclerotic cardiovascular complication is the leading cause of death in diabetic patients [1]. However, the pathophysiological mechanisms of diabetes-associated atherosclerosis remain elusive to date. Among these, endothelial dysfunction induced by hyperglycemia is commonly considered as an initial and crucial event. Specifically, long-term exposure to high glucose causes overproduction of reactive oxygen species (ROS) by endothelial cells, activating nuclear factor kappa B- (NF-*κ*B-) dependent inflammation, decreases nitric oxide (NO) availability, and eventually induces endothelial dysfunction and initiates the development of atherosclerosis [2–4]. Thus, endothelial cell protection against high glucose-induced oxidative injury may serve as a promising therapeutic strategy against diabetic vasculopathy.

Prolyl isomerase-1 (Pin1) belongs to the parvulin family of PPIase enzymes and is first discovered by Professor Lu Kunping in 1996. It is a unique enzyme in humans that is capable of isomerizing specific phosphorylated Ser/Thr-Pro bonds in many substrate proteins, regulating their activity, stability, phosphorylation levels, subcellular localization, and interaction with other proteins [5, 6]. Pin1 regulates protein function following phosphorylation and is traditionally recognized as a key protein that is positively associated with tumorigenesis, while negatively associated with Alzheimer's disease [7]. Recently, it has also been reported that upregulation of Pin1 protein expression and activity was involved in the pathogenesis of diabetes-associated endothelial dysfunction through facilitating mitochondrial translocation of the prooxidant adaptor p66Shc. This eventually promotes the excessive ROS generation and NF-*κ*B-dependent inflammation [8, 9]. On the contrary, Pin1 inhibitor has been proved to be effective in the prevention of mitochondrial oxidative stress and diabetic vascular disease [10].

Vitamin D belongs to fat-soluble secosteroids that provides many skeletal and extraskeletal health benefits. Upon activation of vitamin D receptor (VDR), vitamin D regulates approximately 3% of the human genome [11, 12]. A growing body of evidence has suggested that vitamin D deficiency is associated with increased risk of diabetic vasculopathy, while supplementation of it could effectively protect against diabetic vascular dysfunction through its antioxidant effects [13–15]. However, it still remains unknown how vitamin D exerts its antioxidant effects under hyperglycemic conditions. Our pilot studies have showed that calcitriol, the bioactive metabolite of vitamin D, can inhibit atherosclerosis progression in diabetic *Apoe* ^−/−^ mice partially through the suppression of NAPDH oxidase-derived ROS generation and endothelial apoptosis [16]. However, whether vitamin D can also inhibit Pin1-mediated mitochondrial-derived ROS generation and alleviate endothelial oxidative damage still remains unclear.

Therefore, we explored the study by using Juglone, a potent Pin1 inhibitor, as a positive control. Our study investigated whether vitamin D treatment can inhibit circulatory Pin1 levels and improve endothelial function in diabetic mice at the animal level and whether the endothelial protective effect of vitamin D is associated with its suppressing effects on Pin1-mediated mitochondrial oxidative stress and inflammation at the cellular level, and lastly verified whether the protective effects of vitamin D rely on VDR activation.

## 2. Materials and Methods

### 2.1. Animal

8-week-old male C57BL/6 mice provided by the Shanghai SLAC Laboratory Animal Co. Ltd. were used in all the animal experiments. Diabetic mice were established by intraperitoneally injecting a single dose of streptozotocin (STZ, 150 mg/kg) dissolved in sterile 0.025 M citrate buffer (pH 4.5). The mice were then randomly assigned to the DM group (vehicle, *n* = 10), VitD group (calcitriol 10 *μ*g/kg/d, i.g., twice a week, *n* = 10), or Juglone group (Juglone 1 mg/kg/d, i.p., every other day, *n* = 10). The other 10 C57BL/6 mice were selected as the control group. Mice were housed in temperature-controlled cages (20–22°C), fed ad libitum, and maintained on a 12 : 12 h light/dark cycle. After 8 weeks, blood samples were obtained after a 12 h fast by retroorbital puncture under barbital-induced anesthesia, and serum aliquots were stored at 4°C until analysis. All the experiments were approved by the Animal Ethics Committee of Fujian Medical University and performed in accordance with the institutional guidelines.

### 2.2. Organ Chamber Experiments

Mice were euthanized after administration of sodium pentobarbital (50 mg/kg, i.p.), and their thoracic aortas were isolated and placed immediately in cold modified Krebs–Ringer bicarbonate solution (pH 7.4, 37°C, 95% O_2_; 5% CO_2_) of the following composition (mmol/L): NaCl (118.6), KCl (4.7), CaCl_2_ (2.5), KH_2_PO_4_ (1.2), MgSO_4_ (1.2), NaHCO_3_ (25.1), glucose (11.1), and calcium EDTA (0.026). The aorta was cleaned from adhering connective tissue under a dissection microscope and cut into 2 mm rings for organ chamber experiments. Each ring was then connected to an isometric force transducer (PowerLab ML870 8/30, AD Instruments, Bella Vista, Australia), suspended in an organ chamber filled with 5 mL Krebs–Ringer bicarbonate solution (37°C, pH 7.4), and bubbled with 95% O_2_ and 5% CO_2_. After a 30 min equilibration period, the rings were gradually stretched to the optimal point of their length-tension curve, as determined by the contraction in response to potassium chloride (80 mmol/L). Two rings cut from the same aorta were studied in parallel. Concentration-effect curves were produced by adding graded concentrations of acetylcholine (ACH, 10^−9^ to 10^−5^ mol/L) during submaximal contractions to norepinephrine (10^−5^ mol/L). Relaxations were expressed as percentages of precontracted tension and pD2 values (−1og_10_ EC50) for ACH and interpolated from each curve.

### 2.3. Circulatory Levels of Pin1, SOD, MDA, IL-1*β*, IL-6, and NO

The plasma samples were separated from the blood. The plasma levels of Pin1, interleukin-1*β* (IL-1*β*), and interleukin-6 (IL-6) were detected using enzyme-linked immunosorbent assay (ELISA) kit (Jiancheng Biotech Co. Ltd., Nanjing, China), and the plasma levels of malondialdehyde (MDA) and superoxide dismutase (SOD) were detected by TBA and WST-1 assay, respectively (MDA and SOD kit, Jiancheng Biotech Co. Ltd., Nanjing, China) according to the manufacturer's protocols. NO levels were measured by the Griess method using a nitric oxide detection kit (Enzo Life Sciences) after conversion of nitrate to nitrite as previously described [17].

### 2.4. Cell Culture

Human umbilical vein endothelial cells (HUVECs) are widely used as models for studying the function and pathology of the endothelium. HUVECs were isolated from umbilical cords in accordance with the ethical standards as formulated in the Declaration of Helsinki. Briefly, cells were cultured in 0.1% gelatin-coated flasks (Costar, Cambridge, MA) and grown in a medium (Invitrogen) containing a normal concentration of glucose (5.5 mM). The cells were identified as the endothelium by their morphology and the presence of factor VII-related antigen detected using indirect immunofluorescence. The purity of HUVECs in the culture was higher than 95% and only passages from 1 to 3 were used in the study to avoid age-dependent cellular modifications. For high-glucose treatment, a glucose solution was added up to 33 mM (final concentration).

### 2.5. Pin1 Activity Assay

Pin1 activity was assessed by using a commercially available kit (R&D Systems Inc., Minneapolis, USA).

### 2.6. Detection and Quantification of Intracellular ROS Levels and H_2_O_2_ Production

Dihydroethidium (Sigma, St Louis, MO) can react with O_2_·^−^ in cells to form fluorescent ethidium. HUVECs were incubated in a medium containing dihydroethidium (10 *μ*M) for 30 min at 37°C. Ethidium red fluorescence was detected using Leica TCS-SP2 confocal microscope (Wetzlar, Giessen, Germany). ROS production was measured in 2′,7′-dichlorodihydrofluorescein diacetate- (H2-DCFDA-) treated HUVECs by flow cytometry using Coulter Epics XL (Beckman Coulter, USA). On oxidation, H2-DCFDA becomes highly green fluorescent 2′,7′-dichlorofluorescein. HUVECs were incubated with a medium containing H2-DCFDA (10 *μ*M) for 1 h at 37°C, washed with PBS, and collected. Signals were obtained using a 525 nm band-pass filter (FL1-channel). Detection of HUVEC H_2_O_2_ production was performed with the Amplex Red assay (Invitrogen, Eugene, OR) based on the conversion of Amplex Red (50 *μ*M) into fluorescent resorufin in the presence of horseradish peroxidase (0.1 units/ml) after a two-hour incubation with cells [18].

### 2.7. Apoptosis Assessment

HUVECs were collected and fixed in methanol/acetone solution for 5 min and washed with PBS. Fixed cells were then stained with 0.1 ng/ml Hoechst 33258 (Boehringer Mannheim) for 10 min in the dark to counterstain the nuclei. The cells were observed and photographed under a Nikon fluorescence microscope. In some experiments, the Annexin V/propidium iodide (PI; BD Clontech) was used to quantify the number of apoptotic cells. Cells were washed twice with PBS and stained with Annexin V and PI for 20 min at room temperature. The level of apoptosis was determined by measuring the fluorescence of the cells by flow cytometry analysis.

### 2.8. Cellular Fractionation and Immunoblotting

Mitochondrial protein was isolated by using a mitochondrial fraction kit (Active Motif, Carlsbad, CA). p66Shc mitochondrial translocation was assessed by immunoblotting and analyzed as the percentage change of protein content in the mitochondria versus cytosol. Nuclear protein was obtained by using a nuclear extraction kit (Active Motif, Rixensart, Belgium). NF-*κ*B p65 nuclear translocation was assessed by immunoblotting and analyzed as the percentage change of protein content in the nucleus versus cytosol. GAPDH and COX4 antibodies were used as loading controls for cytosolic and mitochondrial fractions, respectively. For immunoblotting, the corresponding proteins were electrophoresed on 10% SDS-PAGE and transferred onto nitrocellulose membranes. The membranes were blocked with 5% dry milk in PBS-Tween buffer (0.1% Tween 20; pH 7.5) for 60 minutes and incubated with anti-p66Shc (BD Transduction Laboratories™, Franklin Lakes, USA), anti-Pin1, anti-NF-*κ*B p65, anti-caspase-3, anti-eNOS, anti-COX4 (Santa Cruz Biotechnology, Nunningen, Switzerland), and anti-*β*-actin (Millipore, Billerica, USA). Anti-rabbit and anti-mouse secondary antibodies were bought from GE Healthcare (Buckinghamshire, United Kingdom). The immunoreactive bands were detected by an enhanced chemiluminescence system (Millipore, Billerica, USA). Related signals were quantified using a Scion Image Software (Scion Corp., Frederick, USA).

### 2.9. Transfection of Small Interfering RNA

Predesigned small interfering RNAs (siRNAs) against human VDR and controlled scrambled siRNAs were synthesized by GenePharma (Shanghai, China). The dsRNA sequences of VDR and controls used in the current experiments were as follows: for VDR, sense and antisense siRNA were 5′-CAAUCUGGAUCUGAGUGAAdTdT-3′ and 5′-UUCACUCAGAUCCAGAUUGdTdT-3′, respectively. For scrambled control, sense and antisense siRNA were 5′-UUGUCCGAACGUGUCACGUTT-3′ and 5′-ACGUGACACGUUCGGAGAATT-3′, respectively. HUVECs were transfected with 12.5–25 nM oligonucleotide by Transpass R2 transfection reagent (New England BioLabs) under serum-free conditions according to the manufacturer's protocol. After transfection, the cells were incubated with complete medium for 48 h before assaying for target gene inhibition.

### 2.10. Statistical Analysis

Values are expressed as mean ± SEM. Results are compared by one-way factorial ANOVA followed by a post hoc Scheffé comparison test. *P* value < 0.05 was considered to be statistically significant.

## 3. Results

### 3.1. Effects of Vitamin D Treatment on Endothelial-Dependent Relaxation of Thoracic Aorta Isolated from Diabetic Mice

**Figure 1:**
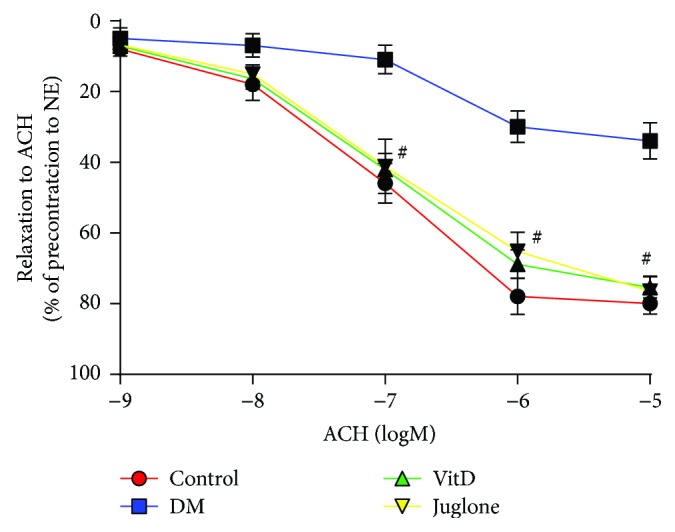
Effects of vitamin D on the endothelial-dependent relaxation of thoracic aorta isolated from diabetic mice. After 1 hour of incubation, the aortic rings were precontracted with norepinephrine (10^−5^ M), and then the rings were exposed to a cumulative concentration of ACH (10^−9^~10^−5^ M) to test the endothelial-dependent vasodilation. Results are mean ± SEM; *N* = 10 each group; ^#^ *P* < 0.05 versus DM.

Compared with control mice, the endothelium-dependent relaxation response to ACH (10^−9^~10^−5^ M) of thoracic aorta isolated from vehicle-treated diabetic mice was markedly impaired. When the concentration of ACH reached 10^−7^ M, the relaxation potential showed significant difference between control mice and vehicle-treated diabetic mice (*P* < 0.05). Both vitamin D and Juglone treatments significantly and equally improved endothelium-dependent relaxation compared with vehicle-treated diabetic mice (*P* < 0.05, Figure 1). The pD2 values in vitamin D-treated and Juglone-treated diabetic mice were significantly higher than those in the vehicle-treated mice (7.00 ± 0.24, 6.97 ± 0.17 versus 6.52 ± 0.32, *P* < 0.05). But no significant difference in pD2 values was observed between the two active treatment groups (*P* > 0.05).

### 3.2. Effects of Vitamin D Treatment on Circulatory Levels of Pin1, MDA, SOD, IL-1*β*, IL-6, and NO of Diabetic Mice

Compared with control mice, the plasma levels of Pin1, MDA, IL-1*β*, and IL-6 were significantly elevated, whereas the plasma levels of SOD and NO were significantly reduced in diabetic mice. Both vitamin D and Juglone treatments significantly decreased the plasma levels of Pin1, MDA, IL-1*β*, and IL-6, as well as reversed the reduced plasma levels of SOD and NO in diabetic mice. Moreover, the plasma levels of Pin1 and SOD of vitamin D-treated diabetic mice were significantly higher than those of Juglone-treated diabetic mice, whereas the plasma levels of MDA of vitamin D-treated diabetic mice were significantly lower than those of Juglone-treated diabetic mice (Table 1).

### 3.3. Effects of Vitamin D Treatment on High Glucose-Induced Apoptosis of HUVECs

**Figure 2:**
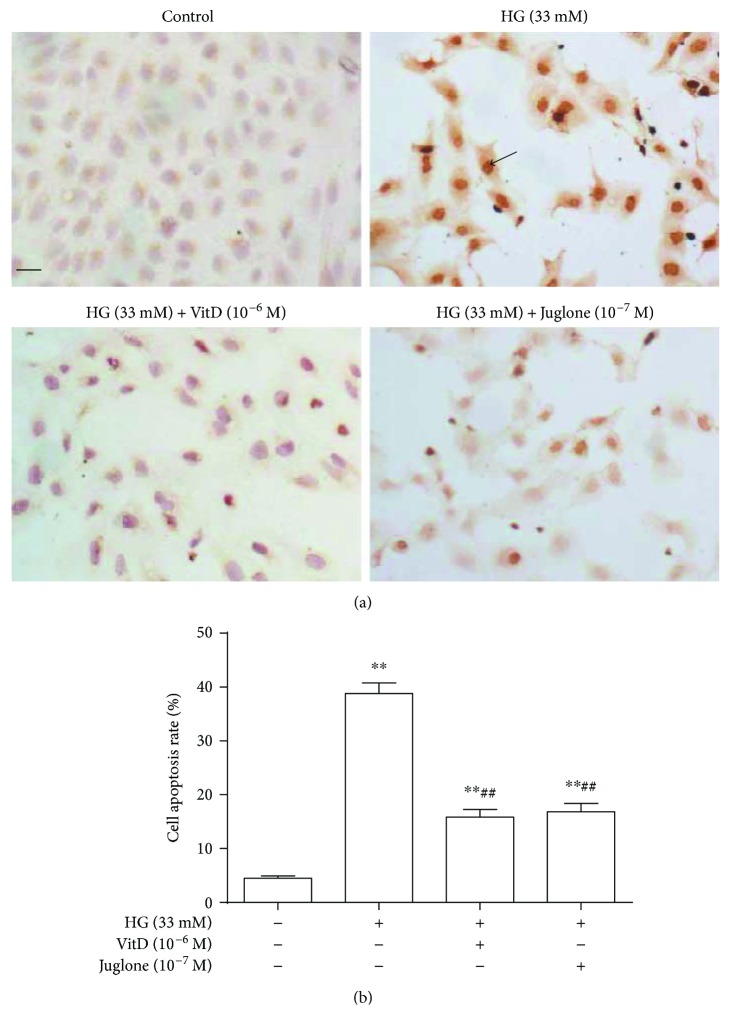
Effects of vitamin D on high glucose-induced HUVEC apoptosis determined by TUNEL method. HUVECs were cultured in petri dishes to 70%~80% confluence and then incubated with high glucose in the presence or absence of vitamin D (10^−6^ M) or Juglone (10^−7^ M) for 72 h. Cells were fixed in 10% paraformaldehyde for 30 min, washed with PBS, and then stained with TUNEL kits for positive apoptotic cells. (a) HUVECs with positive TUNEL staining; scale bar = 10 microns; black arrow indicates positive TUNEL staining. (b) Flow cytometry was used to measure cell apoptosis rate. HG: high glucose (33 mM); results are mean ± SEM (*N* = 5); ^∗∗^ *P* < 0.01 versus control; ^##^ *P* < 0.01 versus HG (33 mM).

Flow cytometry demonstrated apoptosis of high glucose- (33 mM) induced HUVECs in a time-dependent manner. Compared with control HUVECs (mannitol 33 mM), the apoptosis rate of HUVECs induced by high glucose was significantly increased at 24 h and further increased at 72 h but showed no statistical difference at 72 h and 96 h (*P* > 0.05, Supplemental Figure 1(a)). Vitamin D (10^−8^~10^−6^ M) treatment could restrain the apoptosis of high glucose-induced HUVECs in a dose-dependent manner (Supplemental Figure 1(b)). TUNEL staining showed that the control HUVECs arrayed closely, where only a small number of cells with nuclei were stained brown-yellow. Conversely, HUVECs incubated with high glucose for 72 h were arranged loosely, where a large number of cells with nuclei were stained in brown-yellow (Figure 2(a)). Vitamin D (10^−6^ M) or Juglone (10^−7^ M) coincubation remarkably attenuated the apoptosis of high glucose-induced HUVECs (Figure 2(b)).

### 3.4. Effects of Vitamin D Treatment on Pin1 Protein Expression and Activity of High Glucose-Cultured HUVECs

**Figure 3:**
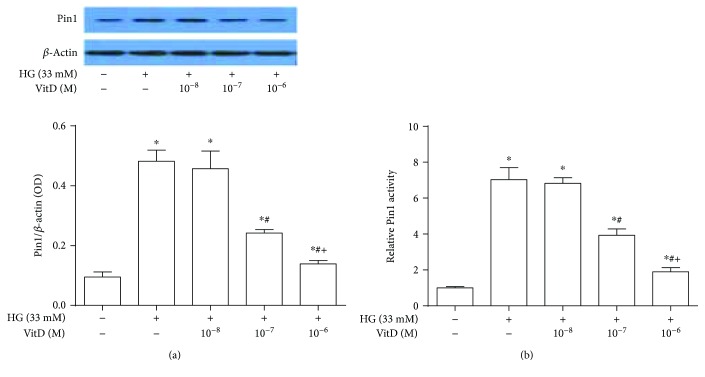
Effects of vitamin D treatment on Pin1 protein expression and activity of high glucose-cultured HUVECs. HUVECs were inoculated in 6-well plates, cultured with 5% FBS at 70%~80% confluence for 24 h, and then coincubated with high glucose (33 mM) and vitamin D (10^−8^~10^−6^ M) for 72 h. Total proteins were extracted after immunoblotting analysis. Pin1 protein expression levels of HUVECs were expressed as the ratio of Pin1 over *β*-actin. Pin1 activity of HUVEC lysate was measured by using a commercially available kit. (a) Effects of vitamin D treatment on Pin1 protein expression of high glucose-cultured HUVECs; (b) effects of vitamin D treatment on Pin1 protein activity of high glucose-cultured HUVECs. HG: high glucose (33 mM); results are presented as mean ± SEM (*n* = 5); ^∗^ *P* < 0.05 versus control; ^#^ *P* < 0.05 versus HG (33 mM); ^+^ *P* < 0.05 versus HG (33 mM) + VitD (10^−7^ M).

Compared with control HUVECs, the Pin1 protein expression and activity of high glucose-cultured HUVECs were significantly increased at 48 h and reached their peaks at 72 h (Supplemental Figures 2(a) and 2(b)). Vitamin D (10^−8^~10^−6^ M) pretreatment for 15 min abrogated the increased Pin1 protein expression and activity of HUVECs under high-glucose condition in a dose-dependent manner. Specifically, the Pin1 protein expression and activity of HUVECs coincubated with vitamin D 10^−7^ M and high glucose were significantly lower than those of HUVECs incubated with high glucose alone and further reduced when coincubated with vitamin D 10^−6^ M and high glucose (all *P* < 0.05, Figure 3).

### 3.5. Effects of Vitamin D Treatment on p66Shc Phosphorylation and Mitochondrial Translocation in High Glucose-Cultured HUVECs

**Figure 4:**
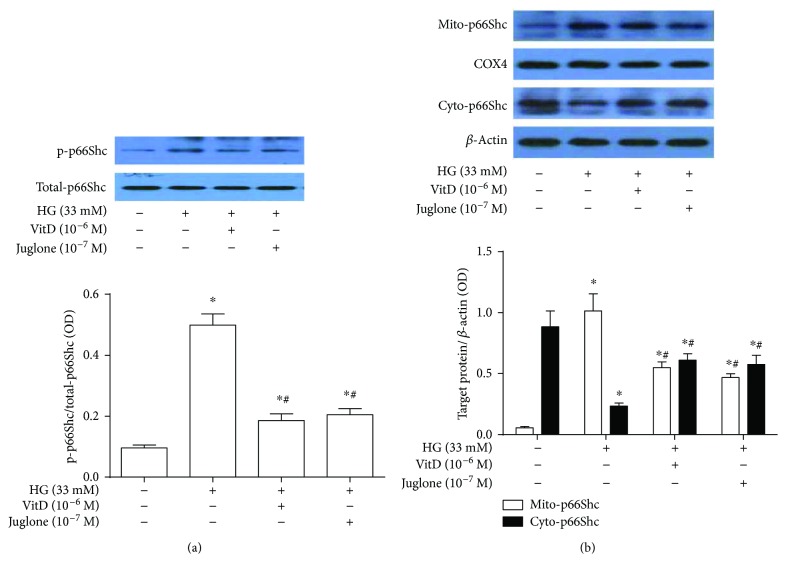
Effects of vitamin D treatment on p66Shc phosphorylation and mitochondrial translocation in high glucose-cultured HUVECs. HUVECs were inoculated in a 6-well plate, cultured with 5% FBS at 70%~80% confluence for 24 h, and then coincubated with high glucose (33 mM) and vitamin D (10^−6^ M) or Juglone (10^−7^ M) for 72 h. Total proteins or cytoplasmic/mitochondrial protein were extracted for immunoblotting analysis. (a) Effects of vitamin D treatment on p66Shc phosphorylation in high glucose-cultured HUVECs. The p66Shc phosphorylation of HUVECs was expressed as the ratio of p-p66Shc over p66Shc. HG: high glucose (33 mM); results are presented as mean ± SEM (*n* = 5); ^∗^ *P* < 0.05 versus control; ^#^ *P* < 0.05 versus HG (33 mM). (b) Effects of vitamin D treatment on p66Shc mitochondrial translocation in high glucose-cultured HUVECs. The mitochondrial and cytoplasmic p66Shc levels were expressed as Mito-p66Shc/COX4 and Cyto-p66Shc/*β*-actin, respectively. HG: high glucose; Mito-p66Shc: mitochondria p66Shc; Cyto-p66Shc: cytoplasm p66Shc; COX4: cytochrome c oxidase subunit 4 (mitochondrial internal protein); results are presented as mean ± SEM (*n* = 3); ^∗^ *P* < 0.05 versus control; ^#^ *P* < 0.05 versus HG (33 mM).

Results showed that vitamin D treatment attenuated high glucose-induced p66Shc phosphorylation at Ser-36 in HUVECs. Specifically, the protein expression levels of p-p66Shc were significantly increased in high glucose-cultured HUVECs compared with control HUVECs. Both vitamin D (10^−6^ M) and Juglone (10^−7^ M) treatments significantly blunted the increased p66Shc phosphorylation in high glucose-cultured HUVECs, and no significant difference was observed between the two active treatments. Unlike Ser-36-phosphorylated p66Shc, there existed no significant difference in the total p66Shc protein expression among the four groups (Figure 4(a)). In addition, high glucose also promoted p66Shc mitochondrial translocation in HUVECs compared with control HUVECs. Specifically, the mitochondrial p66Shc protein levels were significantly increased, while the cytoplasmic p66Shc protein levels were significantly decreased in high glucose-cultured HUVECs compared with control HUVECs (*P* < 0.05). Both vitamin D (10^−6^ M) and Juglone (10^−7^ M) treatments significantly inhibited p66Shc mitochondrial translocation in high glucose-cultured HUVECs but showed no significant difference in their inhibitory effects (Figure 4(b)).

### 3.6. Effects of Vitamin D Treatment on ROS Generation and Caspase-3 Protein Expression in High Glucose-Cultured HUVECs

**Figure 5:**
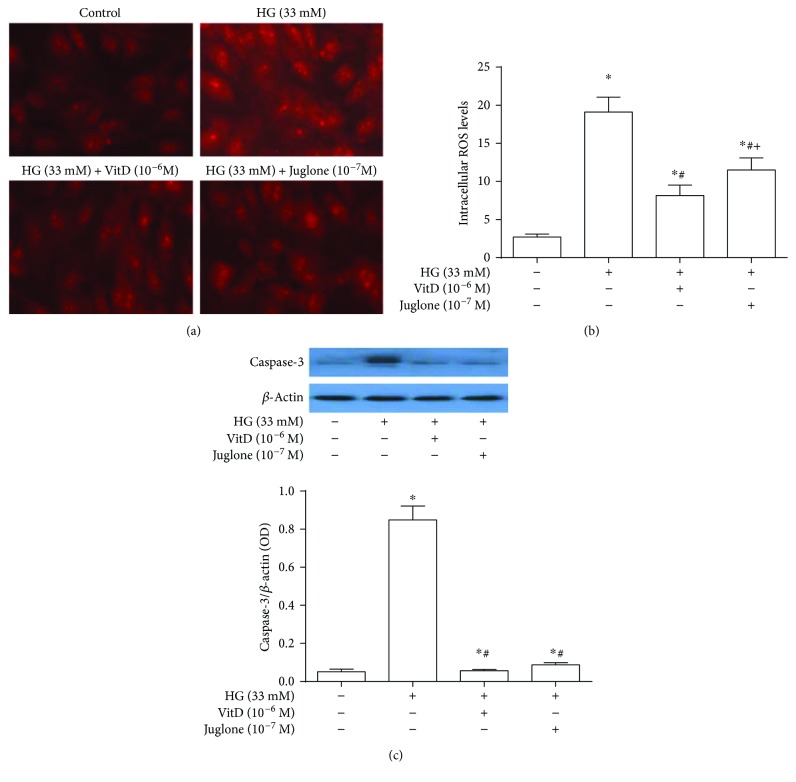
Effects of vitamin D treatment on ROS generation and caspase-3 protein expression in high glucose-cultured HUVECs. HUVECs were inoculated in 6-well plates, cultured with 5% FBS at 70%~80% confluence for 24 h, and then coincubated with high glucose (33 mM) and vitamin D (10^−6^ M) or Juglone (10^−7^ M) for 72 h. (a) The effects of vitamin D treatment on intracellular ROS levels in HUVECs were observed by fluorescence microscope. (b) The effects of vitamin D treatment on intracellular ROS levels in HUVECs were measured by flow cytometry. HG: high glucose (33 mM); results are presented as mean ± SEM (*n* = 5); ^∗^ *P* < 0.05 versus control; ^#^ *P* < 0.05 versus HG (33 mM); ^+^ *P* < 0.05 versus HG (33 mM) + VitD (10^−6^ M). (c) Effects of vitamin D treatment on caspase-3 protein expression in high glucose-cultured HUVECs. Caspase-3 protein expression levels were expressed as the ratio of caspase-3 over *β*-actin. HG: high glucose (33 mM); results are presented as mean ± SEM (*n* = 3); ^∗^ *P* < 0.05 versus control; ^#^ *P* < 0.05; versus HG (33 mM).

Fluorescence microscope revealed a remarkably increased fluorescence intensity of intracellular ROS in high glucose-cultured HUVECs compared with those in control HUVECs. Both vitamin D (10^−6^ M) and Juglone (10^−7^ M) treatments inhibited the red fluorescence intensity in high glucose-cultured HUVECs (Figure 5(a)). Flow cytometry showed that the intracellular ROS levels in high glucose-cultured HUVECs were significantly higher than those in control HUVECs (*P* < 0.05), which were prevented by both vitamin D (10^−6^ M) and Juglone (10^−7^ M) treatments. Further analysis showed that the intracellular ROS levels and H_2_O_2_ contents were significantly lower in vitamin D-treated HUVECs compared to Juglone-treated HUVECs (*P* < 0.05, Figure 5(b) and Supplemental Figure 3, resp.).

Similarly, caspase-3 protein expression levels in high glucose-cultured HUVECs were significantly increased (*P* < 0.05), compared with those in control HUVECs, and were significantly abated by both vitamin D (10^−6^ M) and Juglone (10^−7^ M) treatments (*P* < 0.05). No statistical difference of caspase-3 protein expression levels was observed between vitamin D-treated and Juglone-treated HUVECs (*P* > 0.05, Figure 5(c)).

### 3.7. Effects of Vitamin D Treatment on NF-*κ*B p65 Nuclear Translocation in High Glucose-Cultured HUVECs

**Figure 6:**
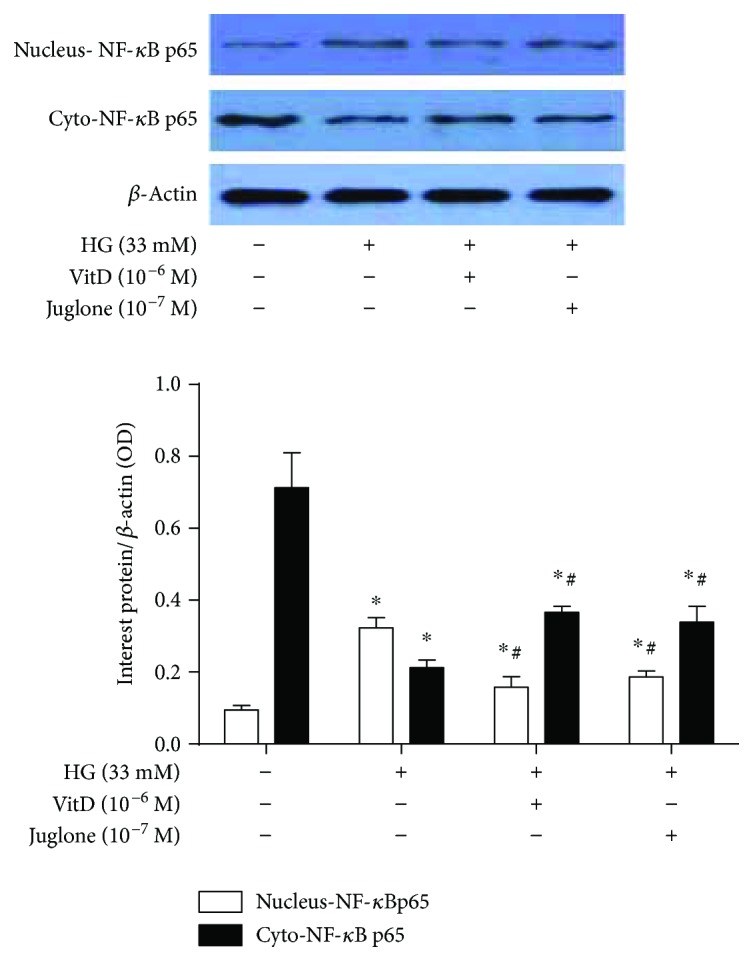
Effects of vitamin D treatment on NF-*κ*B p65 nuclear translocation in high glucose-cultured HUVECs. HUVECs were inoculated in 6-well plates, cultured with 5% FBS at 70%~80% confluence for 24 h, and then coincubated with high glucose (33 mM) and vitamin D (10^−6^ M) or Juglone (10^−7^ M) for 72 h. Total proteins or cytoplasmic/nuclear protein were extracted for immunoblotting analysis. The nuclear and cytoplasmic NF-*κ*B p65 protein levels were expressed as nucleus-NF-*κ*B p65/*β*-actin and Cyto-NF-*κ*B p65/*β*-actin, respectively. HG: high glucose (33 mM); nucleus-NF-*κ*B p65: NF-*κ*B p65 in the nuclear; Cyto-NF-*κ*B p65: NF-*κ*B p65 in the cytoplasm; results are presented as mean ± SEM (*n* = 3); ^∗^ *P* < 0.05 versus control; ^#^ *P* < 0.05 versus HG (33 mM).

Results showed that vitamin D treatment attenuated high glucose-induced NF-*κ*B p65 nuclear translocation in HUVECs. Specifically, the nuclear NF-*κ*B p65 protein levels were significantly increased, while the cytoplasmic NF-*κ*B p65 protein levels were significantly decreased in high glucose-cultured HUVECs compared with control HUVECs (*P* < 0.05). Both vitamin D (10^−6^ M) and Juglone (10^−7^ M) treatments significantly inhibited NF-*κ*B p65 nuclear translocation in high glucose-cultured HUVECs, but no significant difference was observed in their inhibitory effects (Figure 6).

### 3.8. Effects of Vitamin D Treatment on eNOS Protein Expression and Activity and NO Generation in High Glucose-Cultured HUVECs

**Figure 7:**
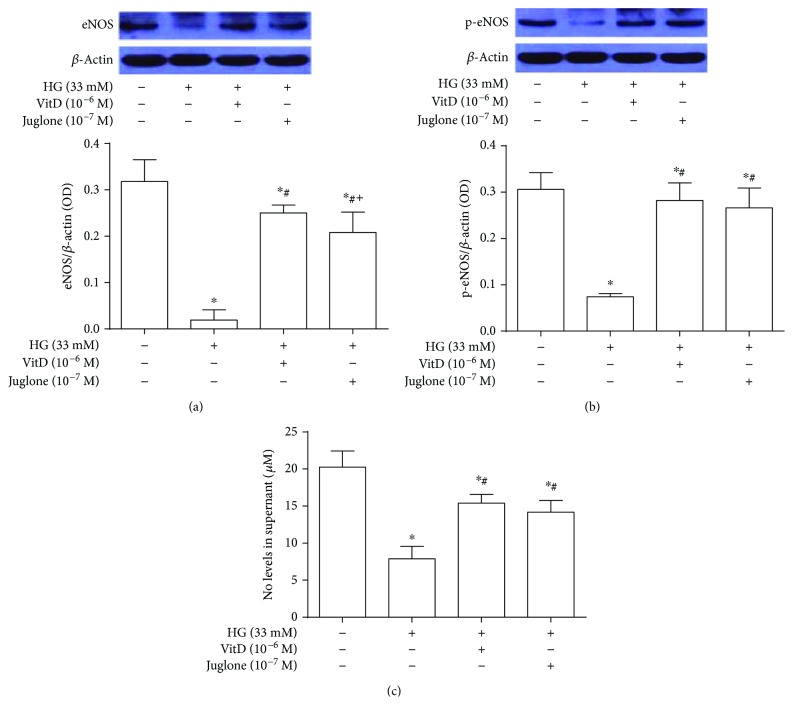
Effects of vitamin D treatment on eNOS protein expression and activity and NO generation in high glucose-cultured HUVECs. HUVECs were inoculated in 6-well plates, cultured with 5% FBS at 70%~80% confluence for 24 h, and then coincubated with high glucose (33 mM) and vitamin D (10^−6^ M) or Juglone (10^−7^ M) for 72 h. Total proteins were extracted for immunoblotting analysis. The eNOS protein expression levels and activity were expressed as the ratio of eNOS over *β*-actin and p-eNOS over *β*-actin, respectively. The NO generation of HUVEC was measured by Griess method. HG: high glucose (33 mM); results are presented as mean ± SEM (*n* = 3); ^∗^ *P* < 0.05 versus control; ^#^ *P* < 0.05 versus HG (33 mM); ^+^ *P* < 0.05 versus HG (33 mM) + VitD (10^−6^ M).

The eNOS protein expression and activity, determined as Ser-1177 phosphorylated eNOS (p-eNOS), and NO generation levels in high glucose-cultured HUVECs were significantly reduced (*P* < 0.05) compared with those in control HUVECs. These were significantly improved by vitamin D (10^−6^ M) and Juglone (10^−7^ M) treatments (*P* < 0.05). The eNOS protein expression levels in vitamin D-treated HUVECs were significantly higher than those in Juglone-treated HUVECs; however, no statistical difference of the eNOS protein activity (p-eNOS) and NO generation levels were observed between vitamin D-treated and Juglone-treated HUVECs (*P* > 0.05, Figures 7(a)–7(c)).

### 3.9. VDR Mediated the Inhibitory Effects of Vitamin D Treatment on Pin1 Protein Expression and Activity in High Glucose-Cultured HUVECs

**Figure 8:**
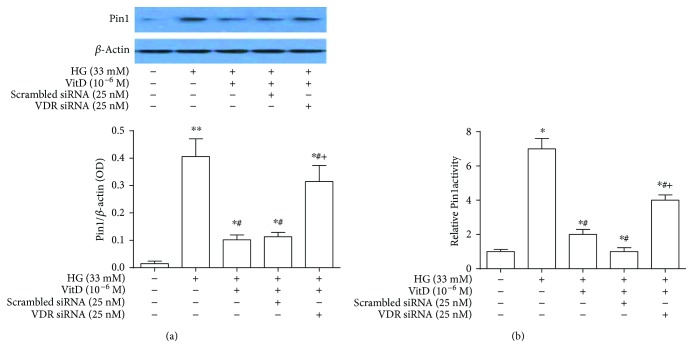
Role of VDR in the inhibitory effects of vitamin D treatment on Pin1 protein expression and activity in high glucose-cultured HUVECs. Total proteins were extracted after VDR-specific siRNA transfection for 72 h for immunoblotting analysis. Pin1 protein expression levels of HUVECs were expressed as the ratio of Pin1 over *β*-actin. Pin1 activity of HUVEC lysate was measured by using a commercially available kit. HG: high glucose (33 mM); results are presented as mean ± SEM (*n* = 3); ^∗^ *P* < 0.05 versus control; ^#^ *P* < 0.05 versus HG (33 mM); ^+^ *P* < 0.05 versus HG (33 mM) + VitD (10^−6^ M) + scrambled siRNA.

VDR-specific siRNA transfection significantly reduced the mRNA and protein expression levels of VDR in HUVECs in a dose-dependent manner. Specifically, the VDR mRNA and protein expression levels were significantly lowered in HUVECs transfected with 25 nM VDR-specific siRNA compared with HUVECs transfected with 12.5 nM VDR-specific siRNA (*P* < 0.05). No significant difference of VDR mRNA and protein expression levels was observed between HUVECs transfected with scrambled siRNA and control HUVECs (*P* > 0.05) (Supplemental Figures 4(a) and 4(b)). Notably, the inhibitory effects of vitamin D treatment on Pin1 protein expression and activity in high glucose-cultured HUVECs were significantly impaired after VDR-specific siRNA transfection (*P* < 0.05). However, no significant difference of Pin1 protein expression and activity was observed after HUVECs were transfected with scrambled siRNA and control HUVECs (*P* > 0.05, Figure 8).

## 4. Discussion

Endothelial dysfunction is generally considered as an initial event in the development of atherosclerosis. Moreover, it has been well demonstrated that high glucose-induced endothelial oxidative injury plays a crucial role in the pathogenesis of diabetes-associated atherosclerosis. Excess ROS production by the endothelium under hyperglycemic condition is mainly derived from NADPH oxidase and the mitochondrial pathway, and the latter accounts for a more proportion [19, 20]. Our prior study has shown that vitamin D can prevent diabetic vasculopathy partially through inhibition of NADHP oxidase-derived ROS generation and endothelial apoptosis [16]. In our current study, we further demonstrated that, for the first time as we know, vitamin D could also protect against diabetes-related endothelial dysfunction through suppressing Pin1-mediated mitochondrial oxidative stress and inflammation and the inhibitory effects of vitamin D on Pin1 expression relies on VDR activation.

ACH is an endothelium-dependent vasodilator that stimulates endothelial cells to release NO and is widely used to evaluate vascular endothelial function. Our organ chamber experiments showed that vitamin D treatment for 8 weeks ameliorated endothelium-dependent relaxation of thoracic aorta in STZ-induced diabetic mice compared to vehicle treatment. Consistent with our findings, Hirata et al. found that 22-oxacalcitriol treatment (0.2 *μ*g/kg) three times per week consecutively for 10 weeks could stop progression of endothelial dysfunction as determined by flow-mediated dilation through antioxidative effects in rats with type 2 diabetes and early-stage nephropathy, without affecting blood glucose levels and blood pressure [14]. In contrast, Salum and his colleagues showed that oral administration of cholecalciferol (500 IU/kg) for 10 weeks failed to improve the functional indices of aortic stiffness as determined by aortic pulse wave velocity or endothelial function as determined by serum levels of asymmetric dimethylarginine [21]. The discrepancy between these results may be attributed to the differences in the selection of vitamin D and different measures used to assess vascular function. Notably, the protective effects of vitamin D against diabetic endothelial dysfunction in our study were comparable with that of Juglone, which prompted us to further explore whether there exists some common mechanisms of action between the two active treatments.

Mechanistically, we found that the endothelial protective effects of vitamin D may be associated with its suppressing effects on Pin1-mediated oxidative stress and inflammation. The increased plasma levels of Pin1 in vehicle-treated diabetic mice versus control mice were significantly attenuated by vitamin D treatment. Moreover, the circulatory levels of oxidative stress and inflammatory indices, as well as the serum levels of NO, were improved by vitamin D treatment. Although the inhibitory effect of vitamin D on plasma levels of Pin1 was obviously weaker than Pin1 inhibitor Juglone, its favorable effects on increasing plasma levels of antioxidant SOD and decreasing plasma levels of ROS byproducts MDA were unexpectedly superior to Juglone. This in turn suggests a more potential antioxidant activity of vitamin D than that of Juglone. Interestingly, this phenomenon was further validated in our in vitro experiment of high glucose-cultured HUVECs. Results suggested that the inhibitory effects of vitamin D on intracellular ROS generation as assessed by flow cytometry significantly outperformed those of Juglone, although its suppressing effects on Pin1 protein expression and activity of endothelial cell was markedly weaker than that of Juglone. The advantage of total antioxidant effects of vitamin D over Juglone may be attributed to the inhibition of NADPH oxidase-derived ROS generation and enhancement of two important transcription factors upregulating the expression of antioxidant enzymes [16, 22, 23]. Lastly, the expression of apoptosis-associated protein caspase-3 in the in vitro experiment showed no difference between vitamin D and Juglone treatments, although a more powerful inhibitory effect of ROS production by vitamin D was observed. The reason for these inconsistencies remains unknown and needs further research.

The p66Shc adaptor protein controls oxidative stress response and life span in mammals. Under hyperglycemic conditions, p66Shc is serine phosphorylated by protein kinase C*β* (PKC*β*) and isomerized by Pin1. This isomerization allows dephosphorylation of Ser-36 residue by serine threonine phosphatase PP2A, inducing the translocation of p66Shc from the cytosol to the mitochondrial intermembrane space (IMS). In the IMS, p66Shc binds to cytochrome c, generating ROS. ROS activates the release of mitochondrial apoptotic factors, eventually inducing apoptosis [24]. Therefore, inhibition of p66Shc-mediated mitochondrial-dependent apoptosis of the endothelium under hyperglycemic condition is especially important for maintaining normal vascular endothelial function. However, relatively less information has been documented concerning the relationship between vitamin D supplementation and mitochondrial-derived ROS generation. Sinha et al. reported for the first time that vitamin D can improve the activity of the mitochondria as well as the muscle function [25]. Uberti and his colleagues demonstrated that vitamin D treatment prevented the loss of mitochondrial potential and the consequently released cytochrome c and activated caspase, attenuating hydrogen peroxide-induced HUVEC apoptosis [26]. However, the underlying mechanisms still remains unclear. Fortunately, our study found that vitamin D can abrogate the increased Pin1 protein expression and activity induced by high glucose, inhibit p66Shc phosphorylation and mitochondrial translocation, and eventually reduce the endothelial apoptosis through the mitochondrial apoptotic pathway. Additionally, both our in vivo and in vitro experiments showed no difference in suppressing inflammation and promoting NO production between vitamin D and Juglone treatments. Why the superior antioxidant effects of vitamin D failed to translate theoretically in suppressing inflammation and improving NO bioavailability still remains unknown. Furthermore, although the eNOS protein expression in the high glucose-cultured HUVECs was upregualted more obviously by vitamin D than by Juglone, the NO production showed little difference between the two active drugs. The disproportionate change of eNOS protein expression and NO production by Juglone may be ascribed to higher NO bioavailability caused by isomerization of the phosphorylated Ser-116 residue in eNOS by Pin1 [9] or reduced iNOS degradation through the calpain pathway [27, 28].

Vitamin D exerts its biological effects by binding to the ubiquitously expressed nuclear VDR and in turn regulates approximately 3% of the genome. Alternatively, vitamin D also exerts its rapid nongenomic actions by binding to membrane VDR [29]. Our in vitro study showed that vitamin D could not only inhibit Pin1 protein expression but also prevent Ser-36 phosphorylation of p66Shc induced by PKC*β* under hyperglycemic condition. Given the fact that the inhibitory effects of vitamin D on PKC activation through the nongenomic pathway has been proved by our previous research, it is presumable that the suppressing effects of vitamin D on mitochondrial oxidative stress were achieved by simultaneous activation of nuclear and membrane VDR. Conversely, knockdown of VDR by siRNA blunted the inhibitory effects of vitamin D on high glucose-induced Pin1 protein expression. All these results suggest an essential role of VDR in mediating the protective effects of vitamin D against mitochondrial oxidative stress, inflammation, and diabetic endothelial dysfunction. Ni et al. demonstrated that the endothelial VDR knockout mice showed impaired acetylcholine-induced aortic relaxation compared with control mice, accompanied by a reduction in endothelial NO synthase expression and increased sensitivity to the hypertensive effects of angiotensin II [30]. Andrukhova et al. also reported that VDR mutant mice are characterized by lower bioavailability of NO due to reduced expression of eNOS, leading to endothelial dysfunction and increased arterial stiffness [31]. These results suggest that endothelial VDR plays an important role in endothelial cell function and blood pressure control, implying a potential role of VDR agonists in the management of cardiovascular disease associated with endothelial dysfunction. Further research is warranted to determine the respective contribution of nuclear and membrane VDR activations in the endothelial-protective effects of vitamin D.

However, there exist some limitations in our research. Firstly, the effects of vitamin D on mitochondrial oxidative stress was not described in detail, such as data on superoxide anion generation by mitochondria, mitochondrial membrane potential, and mitochondrial DNA damage. Secondly, our researches have not explored in detail as to how vitamin D affected Pin1 protein expression and activity. Thirdly, our recent work has reported that vitamin D could abrogate the elevated levels of NADPH oxidase activity and ROS production in HUVEC induced by high glucose; whether this process is also mediated by Pin1 inhibition still remains unknown. Of note, a previous study has proved that Pin1 is critical for tumor necrosis factor-*α*-induced priming of NADPH oxidase and excessive ROS production in human neutrophils [32]. Therefore, a possible interaction between Pin1 and NADPH may also exist in HUVEC under hyperglycemia condition, which needs our further research. Fourthly, further validation of our findings in clinical samples was not done. Therefore, future studies are warranted to further elucidate the role of vitamin D supplementation in the prevention and treatment of diabetic vascular function and its underlying mechanisms.

In conclusion, our research revealed for the first time that vitamin D with the activation of VDR could prevent diabetic vascular dysfunction through inhibiting Pin1-mediated mitochondrial oxidative stress and inflammation. This process was accompanied by reduced Pin1 protein expression and activity and consequently inhibited Ser-36 phosphorylation and mitochondrial translocation of p66Shc, as well as NF-*κ*B nuclear translocation and eNOS protein expression (Supplemental Figure 5). Our research provided new insights on how vitamin D exerts its antioxidant effects and provide novel theoretical basis of vitamin D supplementation in the prevention of diabetic vascular dysfunction.

## Figures and Tables

**Table 1:** Effects of vitamin D treatment on circulatory levels of Pin1, MDA, SOD, IL-1*β*, IL-6, and NO of diabetic mice.

Group	Pin1 (ng/mL)	MDA (nmol/L)	SOD (U/mL)	IL-1*β* (ng/mL)	IL-6 (pg/mL)	NO (*μ*M)
Control	34.3 ± 1.2	7.4 ± 1.0	224.1 ± 35.3	38.1 ± 7.0	64.5 ± 14.2	36.7 ± 3.1
DM	52.3 ± 4.5^∗^	15.7 ± 0.7^∗^	128.5 ± 10.7^∗^	71.9 ± 8.4^∗^	96.4 ± 10.6^∗^	10.2 ± 1.4^∗^
VitD	43.6 ± 3.4^∗^^#^	8.2 ± 1.0^∗^^#^	201.0 ± 21.1^∗^^#^	53.3 ± 9.9^∗^^#^	86.2 ± 17.5^∗^^#^	27.0 ± 2.9^∗^^#^
Juglone	36.8 ± 6.3^#+^	11.7 ± 0.9^∗^^#+^	163.5±22.0^∗^^#+^	53.9±6.4^∗^^#^	83.4±14.4^∗^^#^	25.8±2.7^∗^^#^

Note: *n* = 10 each group; ^∗^ *P* < 0.05 versus control; ^#^ *P* < 0.05 versus DM; ^+^ *P* < 0.05 versus VitD.

## Supplementary Materials

Supplementary Materials Supplemental Figure 1: vitamin D attenuated high glucose-induced apoptosis of HUVECs in a dose-dependence manner. Supplemental Figure 2: high glucose induced upregulation of Pin1 protein expression and activity in HUVECs in a time-dependent manner. Supplemental Figure 3: effects of vitamin D treatment on H_2_O_2_ generation in high glucose-cultured HUVECs. Supplemental Figure 4: effects of VDR-specific siRNA transfection on VDR mRNA and protein expression levels in HUVECs. Supplemental Figure 5: schematic diagram illustrating the proposed mechanisms involved in the endothelial protective effects of VDR agonist under hyperglycemia.
