# Supplementary material for: VDR Agonist Prevents Diabetic Endothelial Dysfunction through Inhibition of Prolyl Isomerase-1-Mediated Mitochondrial Oxidative Stress and Inflammation
Source: Oxid Med Cell Longev. 2018 Apr 15;2018:1714896. doi: 10.1155/2018/1714896 (PMC5925189; doi:10.1155/2018/1714896)
Supplement: Supplementary Materials — Supplemental Figure 1: vitamin D attenuated high glucose-induced apoptosis of HUVECs in a dose-dependence manner. Supplemental Figure 2: high glucose induced upregulation of Pin1 protein expression and activity in HUVECs in a time-dependent manner. Supplemental Figure 3: effects of vitamin D treatment on H2O2 generation in high glucose-cultured HUVECs. Supplemental Figure 4: effects of VDR-specific siRNA transfection on VDR mRNA and protein expression levels in HUVECs. Supplemental Figure 5: schematic diagram illustrating the proposed mechanisms involved in the endothelial protective effects of VDR agonist under hyperglycemia. [file 1714896.f1.pdf]

A

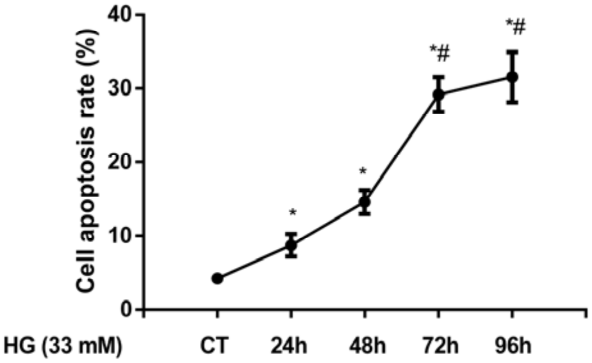

B

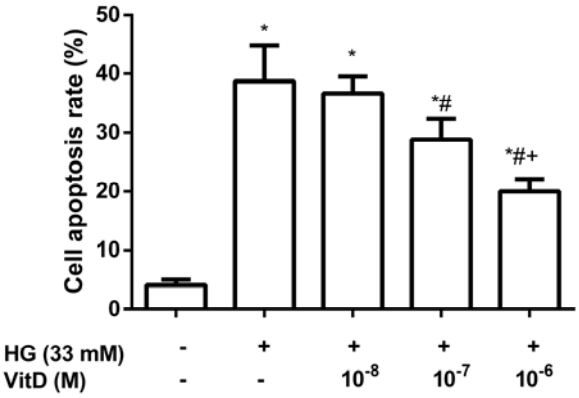

Supplemental figure 1. Vitamin D attenuated high glucose-induced apoptosis of HUVECs in a dose-dependence manner. A: high glucose (33 mM) induced HUVECs apoptosis in a time-dependent manner. HUVECs were inoculated in 9 cm petri dishes, cultured with 5% FBS at 70% ~ 80% confluence for 24 h, and then incubated with high glucose for 24 h, 48 h, 72 h and 96 h, respectively. Flow cytometry was used to detect cell apoptosis rate. HG: high glucose (33 mM), results are mean ± SEM (n= 5), *P < 0.05 *vs* Control, #P < 0.05 vs. 48 h; B: Vitamin D restrained high glucose-induced HUVECs apoptosis in a dose dependent manner. HUVECs were inoculated in 9 cm petri dishes, cultured with 5% FBS at 70% ~ 80% confluence for 24 h, and then incubated with high glucose (33 mM) and different concentrations (10^{-8} ~ 10^{-6} M) of vitamin D. flow cytometry was used to detect cell apoptosis rate. HG: high glucose (33 mM), results are represented as mean ± SEM (n= 5), *P < 0.05 *vs* Control, #P < 0.05 *vs* HG (33 mM), +P < 0.05 vs HG (33 mM) + VitD 10^{-7} M.

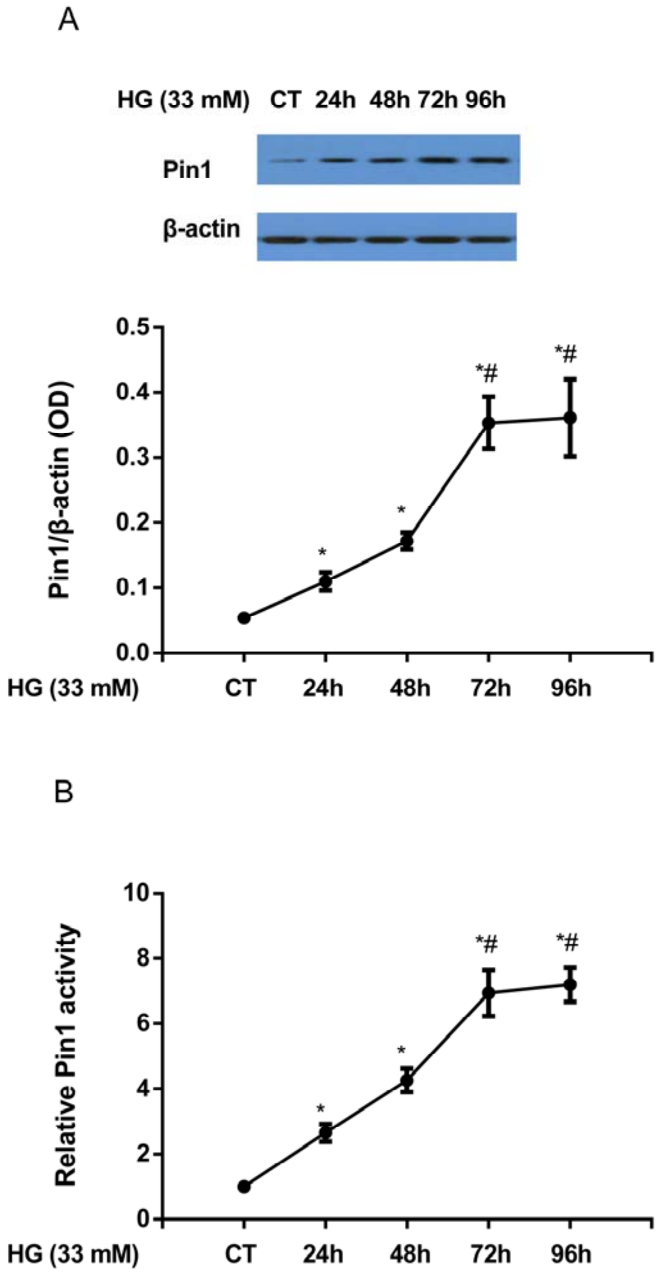

Supplemental figure 2. High glucose induced upregulation of Pin1 protein expression and activity in HUVECs in a time-dependent manner. HUVECs were inoculated in 6-well plate, cultured with 5% FBS at 70% ~ 80% confluence for 24 h, and then incubated with high glucose for 24 h, 48 h, 72 h, and 96 h, respectively. Total proteins were extracted after the intervention for immunoblotting analysis, Pin1 protein expression levels of HUVECs were expressed as the ratio of Pin1 over β-actin, Pin1 activity of HUVECs lysate was measured by using a commercially available kit. A: High glucose induced upregulation of Pin1 protein expression in HUVECs in a time-dependent manner; B: High glucose induced upregulation of Pin1 protein activity in HUVECs in a time-dependent manner. HG: high glucose (33 mM), CT: control, results are represented as mean ± SEM (n = 5), *$P < 0.05$ vs. CT, #$P < 0.05$ vs. 48 h.

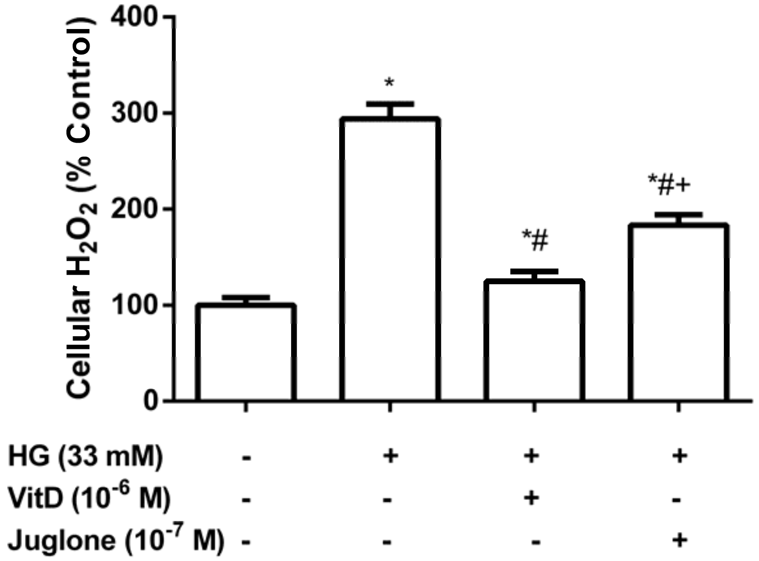

Supplemental figure 3. Effects of vitamin D treatment on H_2O_2 generation in high glucose-cultured HUVECs HUVECs were inoculated in 96-well plates, cultured with 5% FBS at 70% ~ 80% confluence for 24 h, and then coincubated with high glucose (33 mM) and vitamin D (10^{-6} M) or Juglone (10^{-7} M) for 72 h. H_2O_2 was detected with the Amplex® Red Hydrogen Peroxide/Peroxidase Assay Kit. Data are expressed as relative fluorescence in comparison to control. HG: high glucose (33 mM), results are represented as mean ± SEM (n= 5), **P* < 0.05 *vs* Control, #*P* < 0.05 *vs* HG (33 mM), +*P* < 0.05 vs HG (33 mM) + VitD 10^{-6} M.

A

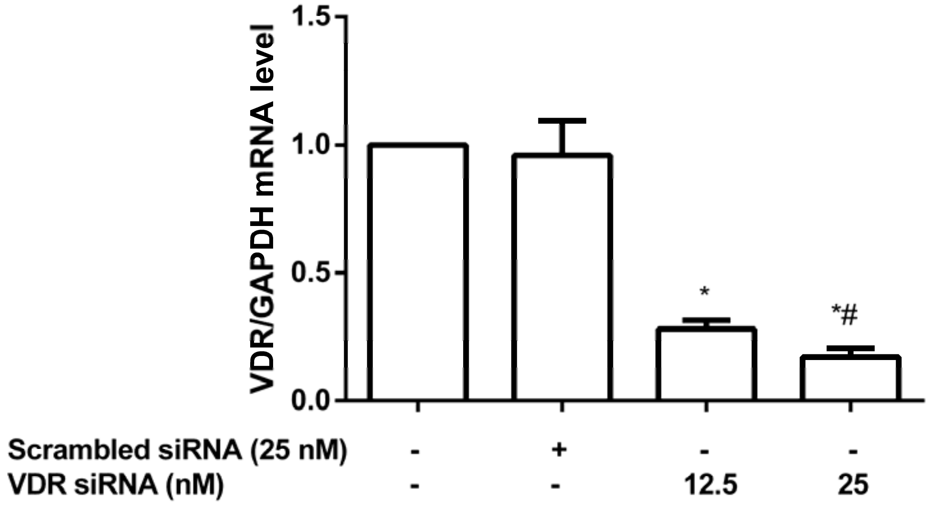

B

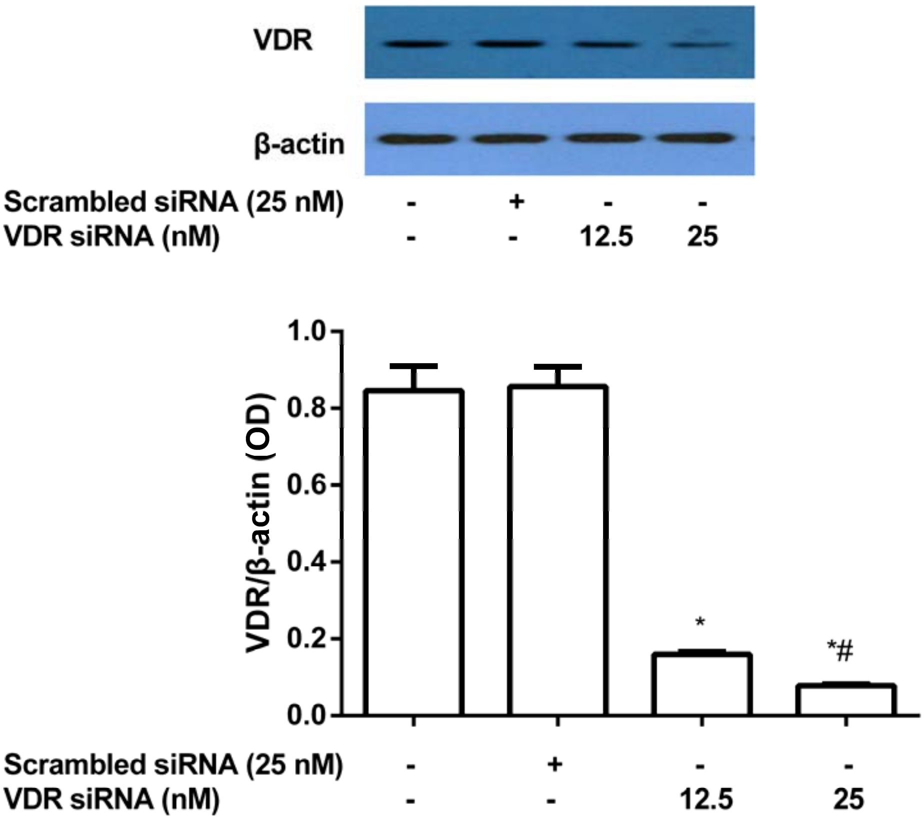

Supplemental figure 4. Effects of VDR specific siRNA transfection on VDR mRNA and protein expression levels in HUVECs. A: Effects of VDR specific siRNA transfection on VDR mRNA expression levels in HUVECs. After VDR specific siRNA transfection for 72 h, the total RNA of HUVECs was extracted and VDR mRNA expression levels were measured by real-time system polymerase chain reaction (RT-PCR). Results are represented as mean ± SEM (n = 3), *P < 0.05, *vs* Scrambled siRNA, #P < 0.05 *vs.* VDR siRNA 12.5 nM. **B**. Effects of VDR specific siRNA transfection on VDR protein expression levels in HUVECs After VDR specific siRNA transfection for 72 h, HUVECs total proteins were extracted and VDR protein expression levels were measured by immunoblotting analysis. Results are represented as mean ± SEM (n = 3), *P < 0.05 *vs* Scrambled siRNA, #P < 0.05 *vs.* VDR siRNA 12.5 nM.

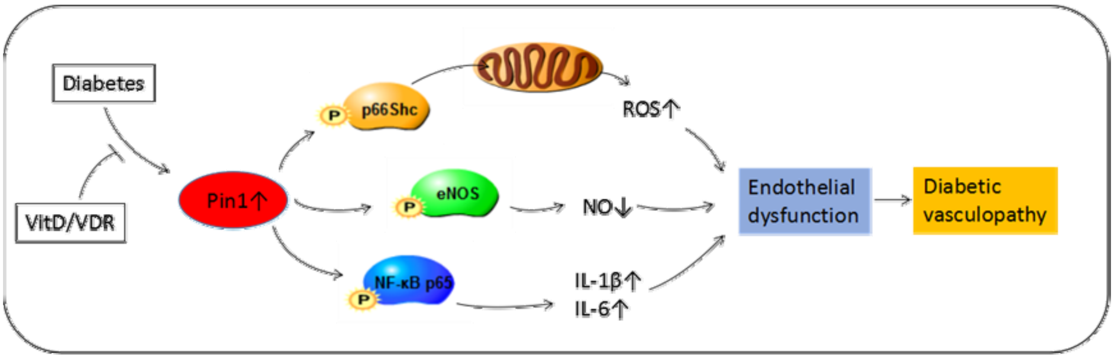

Supplemental figure 5. Schematic diagram illustrating the proposed mechanisms of involved in the endothelial protective effects of VDR agonist under hyperglycemia
